# Roles of sirtuin family members in chronic obstructive pulmonary disease

**DOI:** 10.1186/s12931-022-01986-y

**Published:** 2022-03-21

**Authors:** Xi-Yue Zhang, Wei Li, Jin-Rong Zhang, Chun-Yan Li, Jie Zhang, Xue-Jiao Lv

**Affiliations:** 1 Department of Respiratory and Critical Care Medicine, The Second Hospital of Jilin University, Changchun, Jilin China; 2 Department of Pathogeny Biology, College of Basic Medical Sciences, Jilin University, Changchun, Jilin China

**Keywords:** Sirtuins, COPD, SIRT1, SIRT3, SIRT6

## Abstract

The globally increasing annual incidence of chronic obstructive pulmonary disease (COPD), a common chronic disease, poses a serious risk to public health. Although the exact mechanism underlying the pathogenesis of COPD remains unclear, a large number of studies have shown that its pathophysiology and disease course are closely related to oxidative stress, inflammation, apoptosis, autophagy, and aging. The key players involved in COPD include the sirtuin family of NAD-dependent deacetylases that comprise seven members (SIRT1–7) in mammals. Sirtuins play an important role in metabolic diseases, cell cycle control, proliferation, apoptosis, and senescence. Owing to differences in subcellular localization, sirtuins exhibit anisotropy. In this narrative review, we discuss the roles and molecular pathways of each member of the sirtuin family involved in COPD to provide novel insights into the prevention and treatment of COPD and how sirtuins may serve as adjuvants for COPD treatment.

## Background

Chronic obstructive pulmonary disease (COPD) is a common chronic respiratory disease that is not fully reversible. Currently, COPD is the fourth leading cause of death worldwide, and it is expected to rank even higher by 2030 [1]. COPD affects approximately 10% of individuals over the age of 45, and the disease is equally common among men and women in developed countries [2]. Smoking is a major risk factor for COPD [3]. Moreover, air pollution contributes to the increasing incidence of COPD [4]. The pathogenesis of COPD has not been fully understood. However, owing to its detrimental impact on public health, an increasing number of studies are being conducted on COPD.

Sirtuins are type III histone deacetylases (HDACs), which act on histone proteins present in the chromatin. The sirtuin family consists of seven members (SIRT1–7) in mammals. They exhibit different subcellular localizations in cells and play different roles based on the tissues and cells in which they are expressed. Sirtuins regulate cell metabolism, cell cycle, differentiation, autophagy/apoptosis, and senescence [5]. Owing to the correlation between the pathogenesis (described in the following section) and regulation of sirtuins, there have been many studies on the role of sirtuin family members in the regulation of COPD. Here, we review the roles and molecular pathways of each member of the sirtuin family involved in COPD to provide a reference for the prevention and treatment of this disease.

## Pathogenesis of COPD

COPD is characterized by persistent obstruction in the airways, which is irreversible and progressive, leading to pulmonary ventilation dysfunction, and mainly manifests as chronic bronchitis (goblet cell proliferation and increased mucus secretion) [6] and emphysema (alveolar wall destruction, reduced gas diffusion) [7]. Most of these developments are progressive, leading to a gradual decline in lung function. The etiology of COPD is not yet fully understood, and its origin may be the culmination of long-term interactions between multiple environmental factors and the body’s endogenous factors [8]. Studies have shown that smoking is the greatest risk factor for COPD; however, many people who have never smoked also develop the disease, which may have its origin early in life [9]. Exposure of the fetus or child during infancy to adverse factors (e.g., cigarette exposure, maternal nutrition, birth weight, etc.) may lead to poor lung function [10]. Genetic factors, such as DNA methylation or changes in RNA expression patterns, can also influence the early development of COPD [11]. In addition, exposure to occupational dust, air pollution, infections, immune dysfunction, and aging are factors that lead to COPD [12].

The pathogenesis of COPD is a complex process involving oxidative stress, protease–antiprotease imbalance, and inflammation [13], which is related to the accelerated aging of lung cells [14]. Recent studies have shown that an important mechanism involves the downregulation of HDAC2, which plays an important role in inhibiting the expression of inflammatory genes [15]. Additionally, the endogenous oxidative stress caused by the activation of inflammatory cells in the lung owing to smoke inhalation is an important driving mechanism leading to the reduction in HDAC2 levels [16].

Airway remodeling, characterized by epithelial cell damage, small airway smooth muscle layer thickening, vascular proliferation, gland enlargement, increased mucus secretion, alveolar destruction, inflammatory cell infiltration, and epithelial–mesenchymal transition (EMT), is a characteristic pathological change of COPD [17]. Cigarette smoke stimulates the release and secretion of transforming growth factor (TGF)-β1, causes oxidative stress, and induces the differentiated bronchial epithelium to undergo EMT [18], which can induce airway remodeling and COPD [19].

COPD also accelerates aging of the lungs. Several studies have shown that the number of senescent cells in patients with COPD is increased [12, 20]. The incidence of COPD may be related to the aging of the human population, as the disease mainly affects the elderly, above the age of 65 years. The progression of airway obstruction in patients with COPD is gradual, and the decline in lung function accelerates with age, suggesting that COPD is involved in accelerating the aging process of the lungs [14, 21].

Therefore, it is important to identify the complex pathways involved in the pathogenesis of COPD. Sirtuins, as important anti-aging molecules, are involved in regulating oxidative stress and chronic inflammatory response, and may be involved in the occurrence and development of COPD; these activators may be potential therapeutic targets. From this perspective, sirtuins appear to be important target molecules and will be further discussed in this review.

## Physiological function of sirtuins

The sirtuin protein family is an ancient family of proteins found across all eukaryotes. Sirtuin is an important gene-silencing complex that belongs to HDAC class III [22], and is considered an important regulator of longevity, anti-stress, anti-inflammation, and anti-aging [23]. However, its catalytic mechanism is different from that of other HDACs, as it requires nicotinamide adenine dinucleotide (NAD+) as a cofactor to catalyze the deacetylation of a specific substrate; this occurs by removing the acetyl-lysine of histones to disable ε-acetylamino, thereby inhibiting gene transcription [24, 25].

The sirtuin family was first discovered in yeast [26], and subsequently in higher eukaryotic nematodes [27] and *Drosophila* [28]. Sirtuins are involved in the regulation of multiple pathways, and play important roles in the regulation of DNA damage and pro-inflammatory gene transcription, which are closely related to longevity, apoptosis, and aging [29–31]. There are seven members in the mammalian sirtuin family, namely SIRT1–7 [32–34]. Studies have shown distinct subcellular localizations of each member of the sirtuin family. SIRT3, SIRT4, and SIRT5 are present in the cytoplasm and the mitochondria; SIRT6 is located in the nucleus; SIRT7 is localized in the nucleolus; and SIRT1 and SIRT2 can be found in both the nucleus and the cytoplasm [35, 36]. From the nucleus to the cytoplasm, sirtuins regulate a variety of cellular homeostasis mechanisms, playing different roles depending on the tissue and cell types in which they are expressed. Studies have shown that regulatory genes of the sirtuin family influence a variety of epigenetic factors, which can regulate the average life expectancy of the organism and affect their response to stress and toxicity [37]. Sirtuins also play important roles in other biological aspects such as metabolic diseases, and in the control of the cell cycle, cell proliferation, and aging, which could promote cell survival, apoptosis, or aging [37–39]. All seven members of the sirtuin family play different roles in COPD, which are discussed in further detail in the next section.

## Sirtuins and COPD

### SIRT1

SIRT1 was the first sirtuin discovered in mammals, which is the most widely studied sirtuin and is mainly found in the nucleus but also in the cytoplasm [40]. SIRT1 is an HDAC protein, which deacetylates many key regulatory proteins and transcription factors involved in DNA repair, inflammation, antioxidation, and cell aging, thereby regulating inflammatory responses, oxidative stress, autophagy/apoptosis, and aging [41]. Studies have shown that SIRT1 levels in the lung cells and peripheral blood mononuclear cells of patients with COPD are lower than those in healthy controls, which may be due to the increase in acetylation and the enhancement of inflammatory responses [42]. Therefore, SIRT1 may play an important role in inflammation regulation in the pathogenesis of COPD.

In human monocytes, SIRT1 is activated by Toll-like receptor 4, resulting in the deacetylation of p65/RelB and the termination of nuclear factor (NF)-ĸB-dependent gene transcription [43]. SIRT1 negatively regulates NF-κB, an inflammatory transcription factor, and inhibits gene transcription by deacetylating RELA/p65 [44]. Forkhead transcription factor 3a (FOXO3a) is a transcription factor of the forkhead family, which regulates the expression of multiple genes such as those involved in the cell cycle, differentiation, development, apoptosis, metabolism, autophagy, and longevity [45]. SIRT1 deacetylates FOXO3a, which plays an important role in cigarette smoke extract (CSE)-induced autophagy [46], and enhances the antioxidant response by inhibiting p53, inhibiting cell senescence and the NF-κB pathway, activating proliferator-activated receptor-coactivator-1 (PGC-1α), regulating mitochondrial dysfunction, and promoting cell survival under stress [47]. A recent study has shown that SIRT1 deficiency increases FOXO3 acetylation and exacerbates emphysema in CSE-induced mouse models, while activation of SIRT1 reduces FOXO3 acetylation and improves lung functions [48]. These important biological pathways seem to be involved in the development of COPD [49, 50]. Studies have shown that SIRT1 inhibits STAT3 activation in mouse embryonic cells, and acetylation is necessary for STAT3 activation [51]. As an important inflammatory mediator, STAT3 is involved in the regulation of the occurrence and development of COPD [52]. MicroRNAs are also involved in the regulation of SIRT1 expression and play a key role in oxidative stress and aging. MiR-34a reduces SIRT1 levels by activating the phosphoinositide-3 kinase (PI3K)–mammalian target of rapamycin (mTOR) pathway [53]. In turn, SIRT1 inhibits the mTOR signaling pathway and activates autophagy [54]. In COPD, miR-570 reduces SIRT1 levels by activating the p38 MAPK and activator protein (AP)-1 signaling pathways [55].

In addition, SIRT1 is involved in regulating EMT. Both in-vivo and in-vitro studies have shown that the upregulation of SIRT1 expression can inhibit activation of the TGF-β1/SMAD3 signaling pathway and weaken EMT, suggesting that SIRT1 plays an important role in the prevention of airway remodeling in COPD [56]. Transcriptome data obtained using microarray analysis of human lung fibroblasts, which are important participants in COPD fibrosis and airway remodeling, suggest that the transcription factor sterol regulatory element-binding protein 1 (SREBP1) is the main downstream regulator of SIRT1 in environmental COPD. Exposure of human lung fibroblasts to particulate matter (PM) leads to the activation of the SIRT1-SREBP1-PIR/NLRP3 inflammasome axis by inhibiting SIRT1, which can serve as therapeutic targets for PM-related COPD [57].

### SIRT2

SIRT2 is the only sirtuin protein that exists in the cytoplasm (as its major localization), mitochondria, and nucleus. SIRT2 is involved in the regulation of many physiological processes, including the development of age-related diseases such as type 2 diabetes, cardiovascular diseases, neurodegenerative diseases, and COPD, which are all related to its genetic polymorphism [58–62]. SIRT2 may participate in the aging process via the deacetylation of α-tubulin [42]. In the cytoplasm, SIRT2 deacetylates p65, thereby regulating the expression of specific NF-κB-dependent genes [63].

Currently, the role of SIRT2 in the pathogenesis of COPD is unclear, with very few relevant studies. In a case–control study of COPD, 257 single nucleotide polymorphisms (SNPs) from 16 genes were selected and genotyped, and variations in *SIRT2* were found to be associated with COPD susceptibility [64]. Another case–control study suggested that certain *SIRT2* variants are important risk factors for COPD owing to the promotion of chronic systemic inflammation and oxidative stress [62]. These studies demonstrated that SIRT2 variants may play a role in the occurrence of COPD; however, further studies are required to clarify its biological function in COPD, which will help identify new therapeutic targets.

### SIRT3

Evidence suggests that the impairment of mitochondrial structure and function owing to oxidative stress caused by long-term exposure to inhaled irritants, such as cigarette irritants, plays a key role in the development of COPD [65, 66]. Most mitochondrial proteins are acetylated and act as metabolic sensors, a reversible mechanism linking the metabolic state to mitochondrial function [67]. Acetyl-CoA mediates mitochondrial protein acetylation. SIRT3, SIRT4, and SIRT5 are mitochondrial proteins closely related to mitochondrial function and play an important role in mitochondria-related dysfunction during the progression of COPD.

SIRT3, the main deacetylase and the key regulator of mitochondrial activity, can deacetylate metabolic and respiratory enzymes to regulate mitochondrial function [68]. SIRT3 promotes mitochondrial redox balance by promoting NADPH production and activating manganese superoxide dismutase (MnSOD) to increase the production of reduced glutathione [69]. The lysine residue of MnSOD is deacetylated by SIRT3, which increases the enzymatic activity of MnSOD [70]. In addition, SIRT3 interacts with FOXO3a in the mitochondria, and the overexpression of SIRT3 increases the DNA-binding activity of FOXO3a, thereby stimulating the expression of the MnSOD gene [71]. Therefore, SIRT3 regulates mitochondrial oxidative stress via the upregulation and deacetylation of MnSOD. SIRT3 has also been reported to modulate NF-κB-mediated inflammation [72]. In COPD-related studies, SIRT3 activation may inhibit mitochondrial oxidative stress in airway epithelial cells by upregulation of MnSOD and prevent mitochondrial damage, which may provide a new strategy to slow down or prevent COPD [73].

Skeletal muscle dysfunction is one of the most serious extrapulmonary defects in patients with COPD, influenced by oxidative stress and energy metabolism disorder [74]. Mitochondrial damage plays an important role in the occurrence of skeletal muscle dysfunction during the progression of COPD [75]. SIRT3 is a downstream target of PGC-1α, which regulates the oxidation of mitochondrial fatty acids. The PGC-1α/SIRT3 signaling pathway is important in regulating mitochondrial metabolism and oxidative stress [76]. Studies have shown that curcumin can protect skeletal muscle mitochondria and alleviate skeletal muscle dysfunction by upregulating this signaling pathway in a COPD rat model [77].

### SIRT4

SIRT4 is a key regulator of mitochondrial metabolic enzymes and antioxidant defense mechanisms. SIRT4 is present in the mitochondrial matrix of many organs, including the heart, kidney, brain, liver, and skeletal muscles [78–80]. However, the detailed mechanism underlying its enzymatic activity remains elusive. SIRT4 has ADP-ribosyltransferase [78], lipoamidase [81], deacylase [82], and substrate-specific deacetylase [83] activity. The enzyme activity and substrates of SIRT4 differ in different tissues and cells. For example, SIRT4 exhibits ADP-ribosyltransferase activity and inhibits insulin secretion in pancreatic beta cells [78]. It is also involved in the regulation of lipid oxidation ability in the liver and muscle mitochondrial function [84]. In addition, SIRT4 plays an important role in apoptosis, mitochondrial oxidative stress, and antioxidant response [85].

Thus far, there have been few studies on the role of SIRT4 in COPD. COPD is a complex inflammatory lung disease. Pulmonary endothelial dysfunction and vascular remodeling are closely related to its pathogenesis. Some studies have shown that SIRT4 overexpression can inhibit the expression of VCAM-1 and E-selectin in the inflammation of human pulmonary microvascular endothelial cells induced by CSE and can reduce the adhesion of monocytes to endothelial cells. In addition, SIRT4 overexpression can inhibit the degradation of IκBα, thereby inhibiting the nuclear translocation of NF-κB. However, it is still unclear whether SIRT4 is deacetylated via ADP-ribosylation or the NF-κB pathway. The activation of SIRT4 may contribute to the treatment of COPD-related endothelial inflammation and may serve as an effective target; however, elucidating its mechanism of action requires further research [86].

### SIRT5

SIRT5 is located in the mitochondrial matrix and possesses deacetylase activity, but its activity is weak [87]. In addition, because of its ability to modify the negatively charged acyl group, the protein can be desuccinylated, deacylated, and deglutarated [87, 88]. SIRT5 plays a role in maintaining metabolism and cell homeostasis by regulating the activity of substrates in the metabolic process. SIRT5 can regulate myocardial energy metabolism and maintain the dynamic balance of the heart. SIRT5 participates in the formation and development of lung cancer as a tumor-specific promoter or inhibitor. SIRT5 can inhibit the occurrence and progression of neurodegenerative diseases [89].

Currently, there are limited studies on the role of SIRT5 in COPD. However, SIRT5 is involved in the regulation of metabolism, the stress response, and aging [90], and may, therefore, also be involved in the regulation of COPD. Studies have shown that SIRT5 can promote the deacetylation of FOXO3, thereby inhibiting the apoptosis of lung epithelial cells induced by CSE [91]. Thus, SIRT5 may prove to be a novel therapeutic target for smoking-related COPD.

### SIRT6

SIRT6 is located in the nucleus and possesses deacetylase, ADP-ribosylase [92], and long-chain deacylase [93] activities. SIRT6 plays a key role in regulating DNA damage repair, telomere maintenance, genomic stability, inflammation, and metabolic homeostasis, and is associated with longevity [94].

A decrease in SIRT6 levels is observed in the lung and airway epithelial cells of patients with COPD [95, 96]. There are functional similarities between SIRT6 and SIRT1. SIRT6 is also involved in the regulation of autophagy. Interestingly, unlike SIRT1, SIRT6 inhibits cell senescence by inducing autophagy. SIRT6 overexpression can inhibit the senescence of human bronchial epithelial cells induced by CSE. Studies have shown that SIRT6 overexpression weakens autophagy through insulin-like growth factor (IGF)–Akt–mTOR signaling. SIRT6 is also regulated by miR34a, which activates the PI3K–mTOR pathway and leads to a decrease in SIRT6 expression [53] via a feedback loop. SIRT6 also inhibits the senescence induced by TGF-β via p21 proteasomal degradation and inhibits the secretion of IL-1β to prevent fibrosis [97]. SIRT6 is involved in the regulation of oxidative stress. Upon cigarette smoke stimulation, SIRT6 expression decreases, leading to NF-κB activation, reduction in telomere length, and reduction in the levels of β-catenin, VEGF, and NRF2, further increasing oxidative stress and thus accelerating aging and emphysema [12].

SIRT6 and SIRT1 play important roles in the manifestation of the extrapulmonary symptoms of COPD, such as peripheral skeletal muscle atrophy, a very common occurrence in COPD that seriously affects patients’ quality of life. In patients with COPD presenting with skeletal muscle atrophy, the expression level of phosphorylated H2AX, a molecule promoting DNA damage repair, is lower than that in healthy controls, which is correlated with the significantly lower protein levels of SIRT1 and SIRT6. These sirtuins may play a role in the regulation of diseases by repairing DNA damage and maintaining the stability of the genome [98].

### SIRT7

SIRT7 is the least studied of the seven sirtuins to date. SIRT7 is located in the nucleolus; possesses deacetylase [99], desuccinylase [100], and long-chain deacylase [101] activities; and serves as a key mediator of several cellular activities. SIRT7 has been found to play a role in myocarditis [102], tumorigenesis [103], and apoptosis [104]. However, there is a dearth of studies elucidating the role of SIRT7 in COPD and the underlying molecular mechanisms. Nevertheless, recent research has shown that SIRT7 plays a role in the regulation of DNA damage repair [105], the cell cycle [106], and aging [107], suggesting that it also plays an important role in COPD pathogenesis. Therefore, further research on the role of SIRT7 in COPD is required to make a conclusive claim.

## Potential application of sirtuin agonists in COPD

Thus far, numerous studies have been conducted on SIRT1 agonists with a potential role in the prevention and treatment of COPD. Here, we describe studies published in the recent 5 years on SIRT1 agonists and summarize them in Table 1.

**Table 1:** Molecular targets and functions of SIRT1 agonists

Species	Agonists	Molecular targets	Outcomes
Rat	Melatonin	NLRP3, IL-1β	Attenuate airway inflammation
Rat	Melatonin	CHOP and caspase-12	Attenuate apoptosis and endoplasmic reticulum stress
HBE cells	Melatonin	ORP150	Regulate endoplasmic reticulum stress reaction and autophagy
Rat	Resveratrol	PGC-1α	Antioxidant stress
Mice and HBE cells	Hydrogen sulfide	TGF-β1, Smad3	Reduce oxidative stress, modulate EMT and reduced airway remodeling
Mice and RAW264.7	Nucleosides	NF-κB/p65	Anti-inflammatory
Rat	Curcumin	LC3-I, LC3-II, Beclin 1, CHOP, and GRP78	Modulate autophagy and endoplasmic reticulum stress
RAW264.7	Andrographolide	ERK	Inhibit mitochondrial dysfunction, inflammation, and oxidative stress
HBE cells	Sodium hydrosulfide	ORP150	Regulate endoplasmic reticulum stress reaction and autophagy

NLRP3, NLR-family pyrin domain-containing protein 3; IL-1β, interleukin-1β; CHOP, C/EBP homologous protein; ORP150, oxygen-regulated protein 150; HBE, human bronchial epithelial; H_2_S, hydrogen sulfide; TGF-β1, transforming growth factor-β1; PGC-1α, peroxisome proliferator-activated receptor γ coactivator 1α; LC3, light chain 3; ERK, extracellular signal-regulated kinase

In a study of COPD rats, melatonin reduced airway inflammation by activating SIRT1 to inhibit the NLRP3 inflammasome and IL1β [108]. Melatonin also inhibited endoplasmic reticulum stress and improved alveolar epithelial cell apoptosis by upregulating SIRT1 expression [109]. Resveratrol is found in the peel of red fruit or red wine. It can protect the lungs by activating the SIRT1/PGC-1α pathway, regulating antioxidant enzymes, scavenging free radicals, reducing oxidative stress, and improving mitochondrial function [110]. Other studies have shown that hydrogen sulfide (H_2_S) can activate SIRT1, regulate EMT [56], weaken airway remodeling and mitochondrial dysfunction, and improve alveolar epithelial cell senescence and apoptosis [111]. Nucleosides isolated from *Ophiocordyceps sinensis* may inhibit COPD inflammation by activating SIRT1 to act on the NF-κB/P65 pathway in mice or mouse cells [112]. In rat models, curcumin can improve COPD by activating SIRT1 to regulate autophagy and endoplasmic reticulum stress [113]. Andrographolide may also inhibit extracellular signal-regulated kinase (ERK) signaling by activating SIRT1, preventing COPD mitochondrial dysfunction, inflammation, and oxidative stress [114]. Oxygen-regulated protein 150 (ORP150) is a member of the heat shock protein superfamily; melatonin and sodium hydrosulfide may attenuate apoptosis of human Bronchial epithelial (HBE) cells exposed to CSE by regulating endoplasmic reticulum stress response and autophagy through the SIRT1/ORP150 signaling pathway [115]. These agonists offer new possibilities for the treatment of COPD.

Compared with SIRT1, there is little research on the application of agonists in COPD with respect to other members of the sirtuin family. There have been some preclinical studies on SIRT3 agonists, including honokiol [116], melatonin [117], and berberine [118], which are being evaluated for use in the treatment of Alzheimer’s disease, cardiovascular disease, and non-alcoholic fatty liver disease. However, these agonists have not been tested for the treatment of COPD. Moreover, through study of the mechanism of sirtuins in COPD, it was revealed that sirtuins play important roles in COPD; hence, further study of sirtuin agonists is required such that they may be applied in clinical treatments in the future.

## Conclusions

**Fig. 1:**
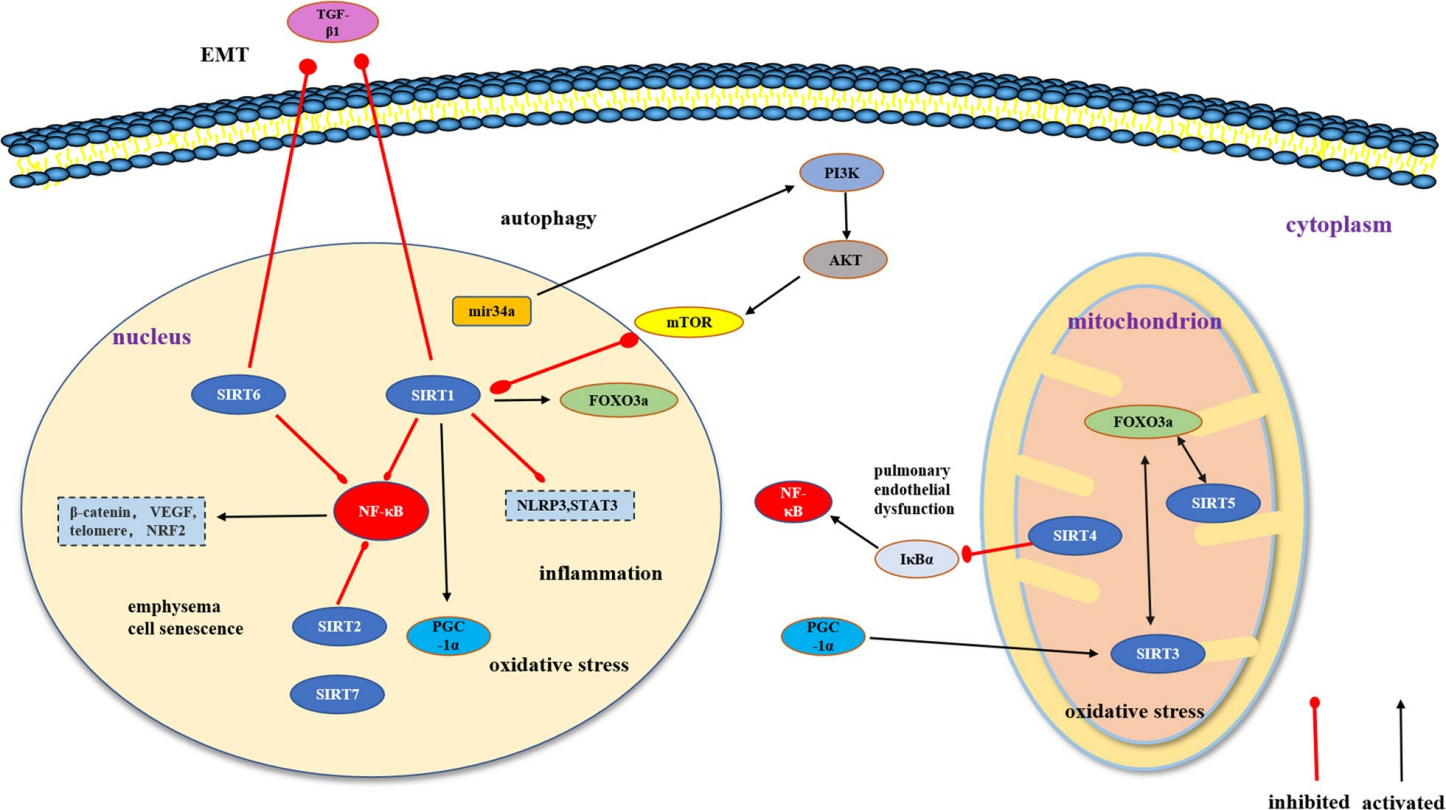
Members of the sirtuin family are involved in the regulation of COPD. Briefly, SIRT1, SIRT2, SIRT6, and SIRT7 are localized in the nucleus. SIRT1, SIRT2 and SIRT6 inhibit the NF-κB signaling pathway to improve inflammatory response, SIRT1 and SIRT6 inhibit the TGF-β1 signaling pathway to alleviate EMT. SIRT1 can also regulate autophagy through the PI3K/mTOR signaling pathway. SIRT3, SIRT4, and SIRT5 are located in the cytoplasm. SIRT3 and SIRT1 improve oxidative stress response by interacting with PGC-1α, SIRT4 inhibits IκBα to improve pulmonary endothelial dysfunction, and SIRT5 and SIRT1 promote FOXO3a deacetylation to regulate apoptosis and autophagy

The sirtuin regulation mechanism of COPD is complex but mainly functions through inhibition of cellular senescence and inflammatory response, reduction of lung endothelial injury and oxidative stress, and regulation of autophagy to improve EMT and emphysema. Each sirtuin family member participates in the regulation of COPD through different pathways (as shown in Fig. 1).

Among the members of the sirtuin family, SIRT1 and SIRT6 are the most widely studied. In particular, SIRT1 and its specific drug activators have been widely studied. However, other proteins in the family have been shown to play a role in the regulation of COPD via different pathways. COPD is highly detrimental to human health. Currently, there is no radical cure for advanced COPD, and the mortality rate is very high. Sirtuins may serve as adjuvants for COPD treatment.

In recent years, the sirtuin family has been extensively studied, leading to some remarkable discoveries. However, there have been very few studies on the impact of sirtuins on COPD. Therefore, it is important to further explore sirtuins as potential therapeutic targets of the disease. Specific drugs such as sirtuin-related agonists may prove to be an effective treatment strategy for COPD. However, owing to the abundant effects of sirtuins, such targeting may lead to potential side effects. Therefore, further studies are required to verify the roles and regulation pathways of sirtuin family members in COPD and to identify suitable targets for the treatment and prevention of COPD.

## Data Availability

Not applicable.

## Abbreviations

COPD: Chronic obstructive pulmonary disease
HDACs: Histone deacetylases
EMT: Epithelial–mesenchymal transition
NAD+: Nicotinamide adenine dinucleotide
TLR: Toll-like receptor
AP: Activator protein
NF-κB: Nuclear factor-κB
FOXO3a: Forkhead transcription factor 3a
HPF: Human lung fibroblast
PI3K: Phosphoinositide-3 kinase
mTOR: Mammalian target of rapamycin
SREBP1: Sterol regulatory element-binding protein 1
PM: Particulate matter
NLRP3: NLR-family pyrin domain-containing protein 3
IL-1β: Interleukin-1β
CHOP: C/EBP homologous protein
ORP150: Oxygen-regulated protein 150
HBE: Human bronchial epithelial
H_2_S: Hydrogen sulfide
TGF-β1: Trans-forming growth factor-β1
PGC-1α: Peroxisome proliferator-activated receptor γ coactivator 1α
MnSOD: Manganese superoxide dismutase
HPMEC: Human pulmonary microvascular endothelial cell
CSE: Cigarette smoke extract
